# Identification of synchronous BOLD signal patterns in white matter of primate spinal cord

**DOI:** 10.21203/rs.3.rs-2389151/v1

**Published:** 2023-03-14

**Authors:** Anirban Sengupta, Arabinda Mishra, Feng Wang, Li Chen, John Gore

**Affiliations:** Vanderbilt University Medical Center

**Keywords:** BOLD, White Matter, Spinal Cord, fMRI

## Abstract

Functional MRI studies of the brain have shown that blood-oxygenation-level-dependent (BOLD) signals are robustly detectable not only in gray matter (GM) but also in white matter (WM). Here, we report the detection and characteristics of BOLD signals in WM of spinal cord (SC) of squirrel monkeys. Tactile stimulus-evoked BOLD signal changes were detected in the ascending sensory tracts of SC using a General-Linear Model (GLM) as well as Independent Component Analysis (ICA). ICA of resting state signals identified coherent fluctuations from eight WM hubs which correspond closely with known anatomical locations of SC WM tracts. Resting state analyses showed that the WM hubs exhibited correlated signal fluctuations within and between SC segments in specific patterns that correspond well with the known neurobiological functions of WM tracts in SC. Overall, these findings suggest WM BOLD signals in SC show similar features as GM both at baseline and under stimulus conditions.

## Introduction

The spinal cord is responsible for the transmission and integration of signals to and from the brain, and acts as the primary interface between the central and peripheral nervous systems^1^. Despite this importance, and in contrast to studies of the brain, there have been relatively few studies of the functional organization of networks within the spinal cord (SC) using state-of-the-art MRI methods. Partly this reflects the technical challenges of imaging the spinal cord due to its smaller physical size, the impact of more pronounced physiological noise, and greater detrimental effects from susceptibility gradients on images^2,3^. Nonetheless, blood oxygenation-level dependent (BOLD) fMRI signals have been reliably detected in the gray matter (GM) of the spinal cord both in response to stimuli and in a resting state. For example, we were among the first to demonstrate robust resting state functional connectivity between the dorsal and ventral horns of the GM of spinal cord in both human subjects and animal^4-6^. Other groups have also reported that a characteristic, reproducible, intrinsic functional architecture is detectable within spinal cord GM^7-10^.

Previous reports of BOLD signals in the cord have focused entirely on GM, even though white matter (WM) comprises 3–4 times the volume of GM in spinal cord^11^. WM BOLD signals from SC have traditionally been considered as nuisance signals and regressed out while analyzing GM^5,9,10^. Functional MRI studies of the brain have also, until recently, rarely reported BOLD signals in WM and WM signals are often used as nuisance regressors in analyzing functional imaging data^12,13^. However, it is clear from our own and other studies that although BOLD effects are weaker in WM, using appropriate detection and analysis methods they are robustly detectable both in response to stimuli and in a resting state in the brain^14-16^. Multiple studies^17-19^ strongly suggest that task-evoked and resting state BOLD effects in WM reflect hemodynamic changes associated with neural activity in cortical areas. It has also been shown that the hemodynamic response of WM in the brain differs from GM and it may vary between regions^20-22^. Moreover, correlations of resting state BOLD signals between different WM tracts and parcellated cortical GM are reproducible and share similarities with those used to infer functional connectivity between GM areas, suggesting that WM BOLD signals reflect the engagement of WM in specific functional networks. Taken together, these findings suggest there is likely value in exploring the nature and organization of BOLD responses and resting state correlations in the WM of the spinal cord too, as a complement to studies of intraspinal GM networks.

Spinal cord WM consists mostly of glial cells and myelinated axons and forms the outer part of the spinal cord surrounding the GM in the center, which is opposite to the organization in the brain^23,24^. The WM is divided into dorsal (or posterior) columns, lateral columns, and ventral (or anterior) columns. These columns are further divided into tracts that carry sensory information up the spinal cord (ascending tracts) and motor information down the spinal cord (descending tracts)^23,24^. Given this anatomic arrangement, the goal of this study was to establish whether BOLD signals are detectable in WM tracts of spinal cord, and to quantify the pattern of responses of WM tracts to external stimuli and their resting state correlations. We acquired functional images of squirrel monkeys which have been previously used as a model to study cervical spinal cord functional connectivity in non-human primates^25-27^. We detected the BOLD signals evoked by sensory stimulation using a conventional General Linear Model (GLM) as well as using a model-free approach and quantified their amplitudes using Fourier transform method. We also detected resting state signals from WM using a data driven approach and analyzed the correlation between signals from different WM regions. Images were acquired with relatively high spatial resolution at high magnetic field (9.4T) to achieve high SNR. The results from this study provide evidence of the presence of BOLD signals in WM of spinal cord, of their synchronous patterns evoked by stimulus and in a resting state and suggest that BOLD signals from WM are relevant for understanding the overall functional architecture of the spinal cord.

## Results

### Stimulus Response in Gray and White Matter of Spinal Cord

**Figure 1:**
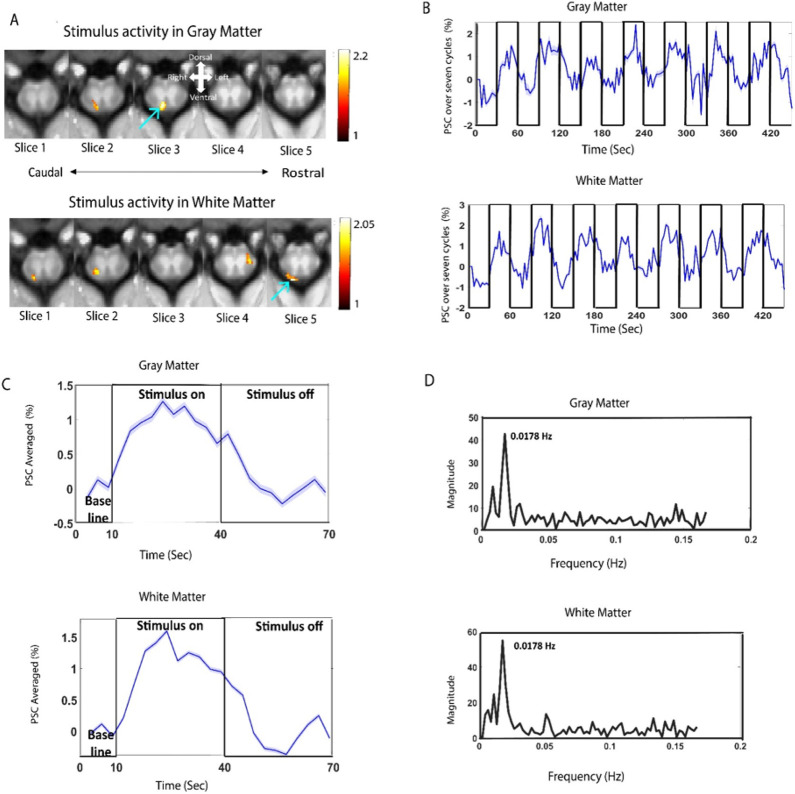
Innocuous tactile (8 Hz vibration) stimuli at digit D2 on left hand evoked fMRI signal changes in the gray matter and white matter of cervical spinal cord of 11 squirrel monkeys (61 runs). **(A)** Group level fMRI T map (thresholded at *t*= 1) overlaid on axial MTC structural images covering C3-C7 segments (corresponding to Slice 5-Slice1). Arrows denote ROI from GM and WM which were used for further analysis. Percentage signal change (PSC) of stimulus evoked fMRI time course at the selected gray and white matter ROI voxels (*t*> 1) over **(B)** the entire stimulus paradigm (baseline 30 secs followed by 7 cycles of 30 sec ‘ON’ and 30 sec ‘OFF) and **(C)** averaged over the 7 cycles of stimulus paradigm. The color shadow in **(C)**around the blue line indicates the +/− standard error of fMRI signal change across epochs. **(D)** Power spectra of the fMRI time courses from the voxels within the selected GM and WM ROIs. The peak frequency is observed at ~0.018 Hz which corresponds to the fundamental stimulus frequency.

Five contiguous axial image slices (3 mm thickness) covering C3-C7 cervical segments, with the third slice positioned over the C5 segment, were acquired in each imaging session and fMRI data was also collected from the same geometry. Supplementary Figure S1 illustrates the locations of the five slices on sagittal and coronal sections and the corresponding transaxial images. Next a stimulus paradigm was designed using a block-design protocol (7 cycles of 30 seconds ‘on’-30 seconds ‘off’ epochs) and corresponding fMRI data were acquired. In response to 8 Hz vibrotactile stimuli on the dominant left hand of 11 monkeys, significant focal fMRI signal changes (t > 1) were detected in the dorsal horns of the GM (slice 2&3) (Fig. 1A). Significant changes (*t*> 1) were also visually observed in the white matter of spinal cord predominantly in the dorsal and spino-cerebellar tracts. Percentage signal changes (PSC) relative to baseline were computed from representative GM and WM region of interest (ROI) shown by arrows in Fig. 1A. The time course of PSC showed robust and synchronous changes in response to the stimulus blocks in voxels of both the GM and WM ROIs (Fig. 1B). The average PSC (from 7 cycles of ‘ON’ and ‘OFF’) was synchronous to the stimulus time course for both GM and WM voxels (Fig. 1C). The maximum PSC within the ROIs reached 1.3% for GM and 1.65% for WM. A total of 32 runs met the t-map threshold (*t*= 1) for the GM ROI and 30 runs for the WM ROI (out of 61 runs). A power spectrum analysis of the fMRI time course showed a distinct peak at 0.018 Hz for both GM and WM voxels, which corresponds, within our frequency resolution, to the stimulus fundamental frequency (1/60 sec) of the task (Fig. 1D). The peak spectral power of WM voxels was found to be higher than those in GM.

The t map from whole spinal cord showing significant responses to the stimulation was thresholded at a lower t value (*t*= 0.8) to evaluate whether the WM voxels were influenced by adjacent GM activation. No such influences were noticed as evident from Supplementary Figure S2 where arrows show that the largest changes in WM regions were not necessarily adjacent to surrounding GM activated voxels.

**Figure 2:**
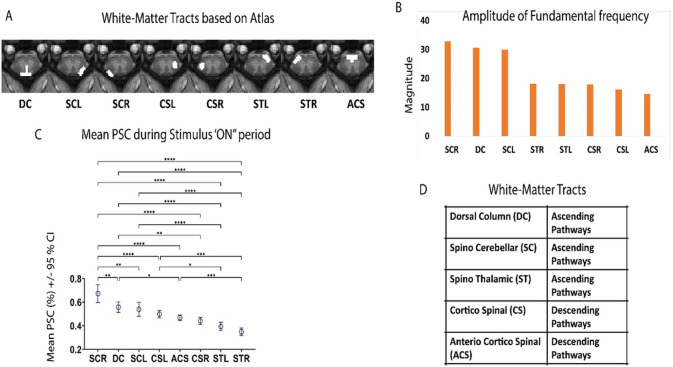
Amplitude of the fundamental frequency of stimulus paradigm and Percentage Signal change (PSC) during ‘stimulus on’ period from different WM tracts of a representative slice (Slice 5) obtained from 11 monkeys (61 runs). (**A)** show the hand drawn white matter tracts based on spinal cord atlas obtained from human and non-human primates. (**B)**Amplitude of fundamental frequency for the stimulus task (~0.018 Hz) obtained from the power spectrum are shown (bar-plots) in descending order for the different WM tracts (**C)**. Mean Percentage Signal Change (PSC) +/− 95 CI (Confidence Interval) during Stimulus On period (averaged over the 7 cycles) and arranged in descending order from the WM tracts. Significantly different values (One-Way ANOVA with Tukey’s multiple comparison test) are denoted by * (*p*<0.05) ,** (*p*<0.01) and *** (*p*<0.01). The established roles of the different WM tracts are specified in (**D)**. Voxels above a particular threshold (*t*>0.5) were selected for the analysis from the WM tracts.

In order to compare the stimulus responses of different WM tracts, amplitudes of the fundamental frequency in response to the stimulus were computed for voxels in hand drawn WM tracts based on a spinal cord atlas obtained from human and non-human primates^24,28-30^. It was found that the spino-cerebellar tracts and dorsal column tract showed higher amplitudes of the stimulus fundamental frequency (0.018 Hz) compared to other WM tracts as shown in the bar plots in Fig. 2B. The mean PSC during ‘stimulus on’ period was also found to be significantly higher (p < 0.05) in spino-cerebellar tracts and dorsal column tracts than most of the other tracts (Fig. 2C).

The canonical hemodynamic response function (HRF) may not be applicable for WM^20-22^ so we also performed Independent Component Analysis or ICA (using 10 components) which is a data-driven, model-free approach on stimulus runs from the WM region in addition to the standard GLM analysis. Certain independent components or ICs (Supplementary Figure S3 A-D) show high positive correlation (*r*> 0.38) with the stimulus paradigm, while other components (Supplementary Figure S3 E-F) show high negative correlations. Thus, the ICA based model free approach also revealed WM signal changes in response to tactile stimuli.

### Independent Component Analysis (ICA) analysis of resting state fMRI data

**Figure 3:**
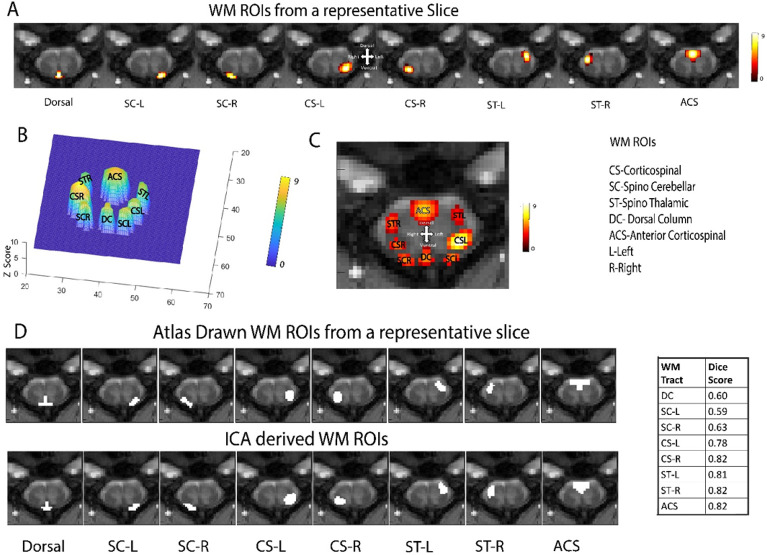
Spatial maps derived from ICA of the WM of spinal cord of 22 monkeys (64 runs) representing 8 regions of interest or hubs. **(A)** Spatial representation of eight independent components from the WM of a representative slice/segment (Slice1/C7). The color bar represents Z-score which was thresholded at Z=4. Each column represents the spatial distribution of one independent component **(B)** 3D plot representing a distinct spatial distribution of the eight WM components (thresholded at Z=5) from the representative slice. **(C)** All the eight WM components overlaid on the representative anatomical image showing the spatial distribution of the hubs. **(D)** Comparisons of location of WM tracts (of a representative Slice 1) derived from Atlas based hand drawn masks (top row) and ICA results (bottom row). The white masked regions overlaid on the MTC images represent the locations of WM tracts in both upper and lower panels. The Dice Score based similarity between Atlas obtained WM tracts and ICA obtained ROIs is provided in the table to the right.

ICA has the advantage of not requiring prior hypotheses about the location of functional responses within any given structure and hence we chose it for unearthing functional circuits in the GM and WM of spinal cord. ICA of resting state fMRI (rsfMRI) data from 22 monkeys (64 runs) revealed the presence of seven components in GM located at spatially distinct ROIs within a slice: left/right dorsal (LD&RD), left/right ventral (LV&RV) horns, left/right intermediate (LI &RI) region which resides between the dorsal and ventral horns, and gray commissure (GC) region, as visible from the spatial distribution of each IC from a representative slice in Supplementary Figure S4. From the WM region, eight regions/hubs of spontaneous BOLD fluctuations were observed from individual slices (most of the hubs were reliably detected in all slices) as shown in Fig. 3A and this corresponded closely to anatomically identified locations of WM tracts in SC as shown later in Fig. 3D. These were the dorsal column (DC) tract, anterior cortico-spinal (ACS) tract, cortico-spinal (CS) tract (left and right), spino-thalamic (ST) tract (left and right) and spino-cerebellar (SC) tract (left and right) (Fig. 3A).

These ROIs were distinctly resolved spatially within the spinal cord WM at a particular threshold (Z > 5), as illustrated in a 3D mesh plot in Fig. 3B and Fig. 3C where the components from one slice are shown together. These ROIs represent clusters of voxels that show temporal synchrony in BOLD fluctuations independent of other clusters, though their extent may also include spatial blurring effects intrinsic to the BOLD mechanism and imaging unsharpness^31^. Supplementary Figure S5 presents a detailed description of the spatial locations of the eight components from each of the five slices.

For testing the spatial specificity of the WM ICs, we compared their spatial locations (thresholded at Z = 4) to those obtained from hand drawn WM tracts based on a spinal cord atlas obtained from human and non-human primates^24,28-30^ (Fig. 3D). The close similarity in their spatial locations is visually evident. A quantitative analysis using Dice scores revealed a similarity of 0.60 for DC, 0.59 for SC-L, 0.63 for SC-R, 0.78 for CS-L, 0.82 for CS-R,0.81 for ST-L,0.82 for ST-R and 0.82 for ACS for that slice.

We also tested the reliability of the estimated ICs using GIFT software’s ICASSO method^32^. The reliability of the ICs was found to be high with a stability index higher than 0.8 for 43 of the 45 components (Supplementary Figure S6A). Supplementary Figure S6B shows the 2D projection of the estimated clusters. Most of the estimated clusters showed high intra-cluster similarity (> 0.9) which is also evident through their compact spatial profile (Supplementary Figure S6B).

For ICA decomposition of GM, we chose 35 components from the 5 slices based on our recently published study^26^. For WM, we chose 45 components from the 5 slices based on anatomical knowledge of WM tracts and empirical evidence of synchronized BOLD fluctuations in specific regions, the details of which are provided in supplementary material. We also ran WM ICA with lower numbers of components to study which, if any, components merge together, because it is known that weaker sub-networks may combine and be represented by a single independent component^33,34^. From the results we can see that at a sufficiently reduced threshold, certain components reveal their presence across multiple segments in similar positions (Supplementary Figure S7B and S7D). Also, certain bilateral WM tracts merge into single independent component (Supplementary Figure S7A and S7C).

### Functional connectivity of WM and GM hubs within and between segments in resting state

The spatial location of each component obtained from ICA was considered as a ROI and correlation measures were computed between their fMRI time-courses. A connectivity matrix showing average correlation (absolute values) between pairs of WM and GM ROIs (obtained using ICA) from 22 monkeys (61 runs) was obtained and many of the correlations were found to be significant (*p* < 0.05) after FDR correction, the details of which are shown in Supplementary Figure S8.

**Figure 4:**
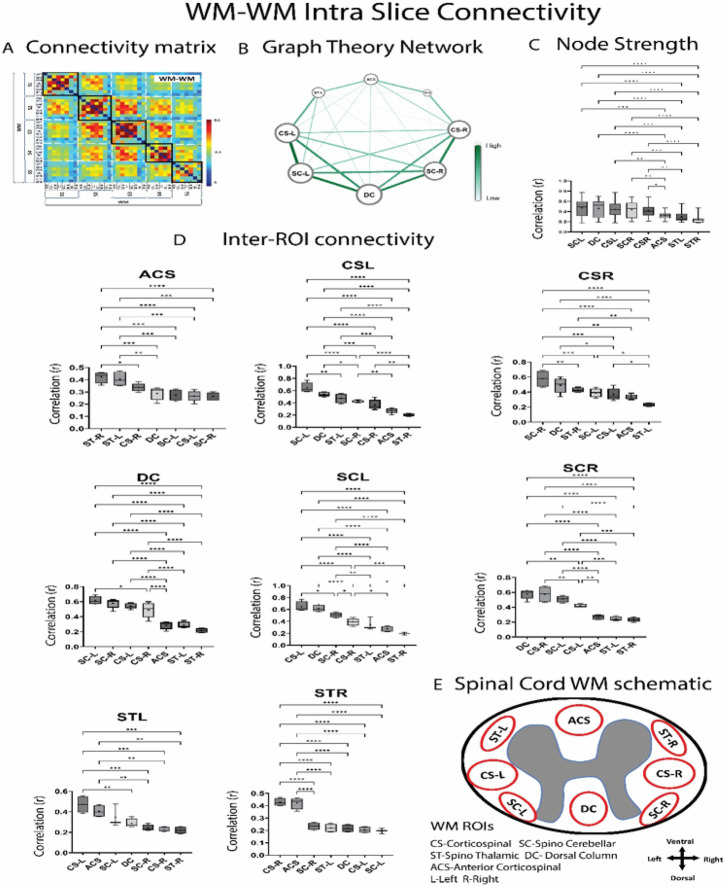
Intra-slice/segment WM-WM connectivity averaged over 22 monkeys (64 runs). **(A)** Correlation matrix (*r* values from Pearson correlation) obtained from 37 WM ROIs covering Slice 1-Slice5 (C7-C3 segment). Intra-slice correlations are shown in the square black boxes near the diagonal. The color bar represents *r* value. Graphical representation of the WM-WM correlations within a slice averaged over the 5 slices are shown in **(B).**Nodes represent the WM ROIs while edges represent functional connectivity values between them. Nodes which have higher node strength (sum of the weight of the links connected to each node) appear bigger while edges which have higher correlation (r) appear darker green. Labels of the nodes are proportional to the node strength. Boxplots depicting the weight of the links of each WM hub that make up its node strength, are arranged in decreasing order and shown in **(C). (D)** Correlation value (r) of each WM hub with others, from the 5 slices are shown using boxplots. Within box-plots, the dashed line represents median value of the distribution and the * represents mean value. Significantly different box-plots (One-Way ANOVA with Tukey’s multiple comparison test) are denoted by * (*p*<0.05) ,** (*p*<0.01) and *** (*p*<0.01). **(E)** is a spinal cord schematic showing the approximate location of the different WM hubs. Abbreviated names of WM ROIs are shown at the bottom. Note all WM hubs were not observed from all segments.

Figure 4A shows the WM-WM connectivity matrix and the intra-slice WM-WM correlations are shown in the black boxes near the diagonal. In general, intra-slice WM-WM correlations between the neighboring WM hubs were found to be significantly higher than those far away, as evident from the inter-ROI connectivity boxplots in Fig. 4D. Thus, for example we see significantly higher correlation of DC hubs with SCL/SCR hubs than those hubs which are further away. A simple network analysis was performed on the connectivity matrix using the Gephi software^35^ based on graph theory principles^36^. The graph plot in Fig. 4B also shows stronger WM-WM connectivity (averaged over slices) among neighboring hubs (thicker edges) compared to the those far away anatomically (thinner edges). Also, the mean of the node strength values was found to be significantly (*p* < 0.05) stronger on the dorsal (DC, SC-L, SC-R) and dorso-lateral region of spinal cord (CS-L, CS-R) than those on the ventral (AC-S) and ventro-lateral side (ST-L, ST-R) as evident from their bigger node sizes in the graph plot (Fig. 4B) as well as from the boxplots in Fig. 4C.

**Figure 5:**
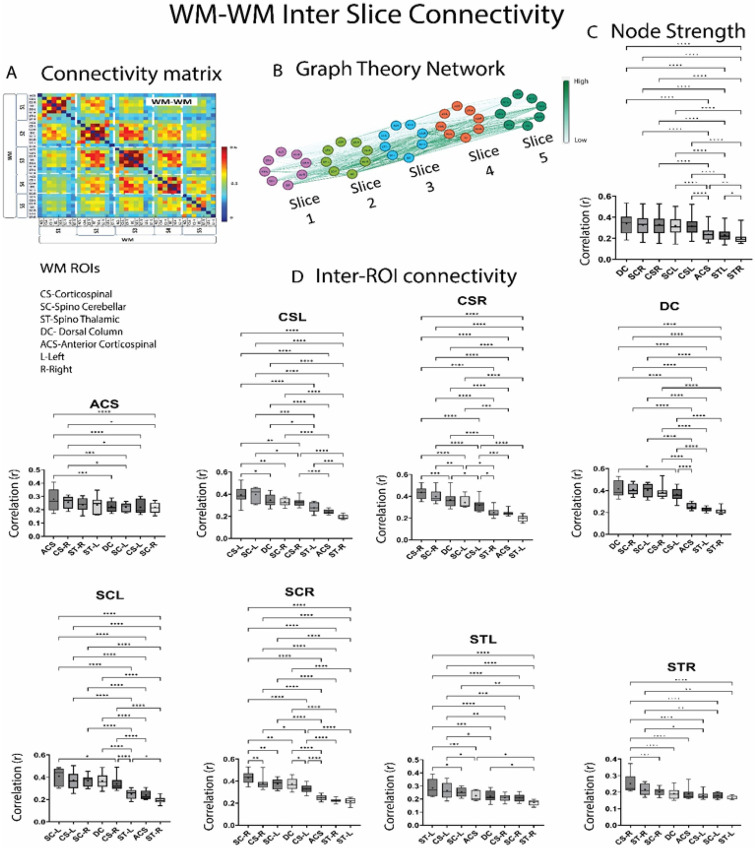
Inter-slice/segment WM-WM connectivity averaged over 22 monkeys (64 runs). **(A)** Correlation matrix (*r* values from Pearson correlation) obtained from 37 WM ROIs covering Slice 1-Slice5 (C3-C7 segment). Inter-slice correlations are arranged vertically in the matrix separated by white dashed lines. The color bar represents *r* value. Abbreviated names of WM ROIs are shown below it. Graphical representation of the inter-slice WM-WM correlations over the 5 slices are shown in **(B).**Nodes represent the WM ROIs while edges represent functional connectivity values between them. Edges which have higher correlation (r) appear darker green. Boxplots depicting the weight of the links/edges of each WM hub that make up its node strength (sum of the weight of the links connected to each node), are arranged in decreasing order and shown in **(C). (D)** Correlation value (r) of each WM hub with WM hubs from other 5 slices are shown using boxplots. Within the box-plots, the dashed line represents median value of the distribution and the * represents mean value. Significantly different box-plots (One-Way ANOVA with Tukey’s multiple comparison test) are denoted by * (*p*<0.05) ,** (*p*<0.01) and *** (*p*<0.01).

Figure 5A shows the inter-slice WM-WM correlations arranged vertically in the connectivity matrix separated by white dashed lines. The mean correlation of each WM hub appears to be maximum with the same WM hub from other slices viz. the strongest correlation of DC from any given slice is with DC of other slices and this is significantly higher than those further away as evident from the inter-ROI connectivity boxplots in Fig. 5D. The mean of the node strength values (Fig. 5C) obtained from the graphical representation of inter-slice WM-WM correlations (Fig. 5B) shows that dorso-lateral and dorsal nodes have significantly (*p* < 0.05) higher value than the ventral/ventro-lateral ones. Similar analysis was also done on WM-GM correlations (Supplementary Figure S9) and GM-GM correlation (Supplementary Figure S10) the results of which are discussed in Supplementary material.

In order to account for potential confounds in correlation values resulting from vascular drainage effects, we performed supplementary analyses to evaluate the effect of regressing out the fMRI time courses of individual WM and GM ROIs on the overall correlations between the ROIs. We found that regressing out the mean signal from a particular hub (both GM and WM hubs) reduced its connectivity significantly with others. We also saw that regressing out signal from a WM hub can change connectivity between GM hubs or between GM and WM hubs not only near it but also distant to it (Supplementary Figure S11) viz. regressing out ACS not only reduces the connectivity between neighboring GM hubs LV and RV but also reduces correlation significantly (p < 0.05) between distant GM ROIs LI and RI from 0.42 to 0.32. Also, correlation between distant GM and WM ROIs LI and CSL reduces significantly from 0.52 to 0.46.

## Discussion

### BOLD signal changes were produced in ascending white matter tracts by tactile stimuli

Increase in BOLD signal in response to tactile stimuli was observed in the dorsal horns of GM of spinal cord confirming previous findings of sensory activation in monkeys^6,27^. Voxels from WM ROI also showed signal increases with tactile stimuli in a manner synchronous with the stimulus paradigm and similar to GM voxels (Fig. 1).

The findings of signal changes within WM mimic those of GM but caution must be shown in interpreting these effects in the same way. The vasculature of spinal cord segments includes multiple smaller veins that drain radially into circumferential larger vessels^37-40^. The WM of spinal cord receives blood supply from arteries outside the spinal cord but is also penetrated by veins carrying blood from the central spinal cord GM. While blood to the GM is supplied by vessels that branch out from the center of the cord, WM arterial supply also enters from the periphery. This implies that in theory, changes in activity within GM could produce BOLD effects in adjacent WM through the veins that drain them and vice-versa. However, our analysis showed that activations in WM regions were not necessarily driven by nearby GM activated voxels which shows that drainage effects are not enough to explain WM activations. The flow to GM is about 4–5 times that of WM in the cord^41,42^ but the volume of WM to GM is also about the same^11,43^. The maximum PSC in WM of spinal cord was found to be slightly higher than the GM, which is contrary to findings in the brain^44^. The energy requirements for WM functions are usually considered low compared to GM^45^, but our results suggest that BOLD signal changes are similar or greater in WM than GM. This phenomenon could be attributable to the presence of larger vasculature in WM of spinal cord. Alternately, these BOLD changes may reflect an intrinsic increase in flow and metabolism within the WM, not necessarily meeting the same requirements as neural activity in GM but perhaps instead reflecting the need for increased flow to meet glial cell or other requirements in WM.

WM activations were visually observed primarily at the dorsal and spino-cerebellar tracts both using GLM, and ICA based methods as shown in Fig. 1 and Supplementary Figure S3. The amplitude at the stimulus frequency was highest in the spino-cerbellar tract followed by DC and the spino-thalamic tracts (Fig. 2B). This can be explained by the fact that dorsal column, spino-cerebellar tracts and spino-thalamic tracts are ascending pathways involved in sensory transmission of stimulus-evoked signals contrary to bilateral and anterior corticospinal tracts which act as descending pathways^24^. The use of a canonical HRF in a GLM is likely not optimal for detecting WM signal changes as in the brain it has been shown to be delayed and different compared to GM^20-22^. Hence, we verified our observations using data driven ICA and our results suggest that the evoked BOLD response in WM may be correlated and sometimes anti-correlated to the stimulus pattern. The negative correlations reflect delays in hemodynamic changes which have been also found in brain WM^21,22^.

### Spontaneous BOLD fluctuations detected in GM and WM during resting state

Our previous work demonstrated the presence of 7 functional hubs within the GM of spinal cord of 5 monkeys using ICA^26^. In this work we replicated the same 7 hubs by performing ICA on a larger dataset of 22 monkeys. Using similar techniques and pre-processing pipeline we also found 8 major components within the WM of spinal cord. To minimize potential signal contaminations from nearby structures, the WM and GM volumes were masked and analyzed separately while performing ICA using GIFT software.

Each component from the ICA decomposition represents voxels showing correlated patterns of BOLD signals overtime, and in GM may be interpreted as functional hubs. The WM hubs were obtained using a model-free and data-driven ICA technique and represent intrinsic patterns of BOLD signal changes in spinal cord. The high stability and intra-cluster similarity of the ICs using the ICASSO method^32^ confirm these WM hubs are robust and reproducible. Each hub was assigned to a corresponding WM tract by comparing the location of its peak value and we found substantial spatial similarity (Dice Score) with anatomical locations of known WM tracts. Thus, it may be inferred that WM tracts in spinal cord that provide structural connectivity also exhibit synchronized BOLD fluctuations in a resting state.

Reducing the number of components using GIFT and lowering the Z-score threshold enabled us to visualize synchronized BOLD fluctuations of some of the WM hubs across segments, which provides further evidence of the anatomical connections of WM tracts across segments (Supplementary Figure S7). Also, it revealed certain components which were bilateral/symmetrical within a segment which is consistent with the known bilateral locations of WM tracts.

### Functional Connectivity of WM hubs followed patterns consistent with their neurobiological functions

The cervical spinal cord WM consists of ascending and descending pathways. The ascending pathways (eg. dorsal column, bilateral spino-cerebellar and spino-thalamic tract) carry sensory information like vibration, conscious proprioception, and fine touch from the body periphery up the spinal cord to the brain. Descending pathways (e.g., Lateral and Anterio-Cortico-Spinal tracts) carry motor information from the brain down the spinal cord to the body. The WM hubs were found to be significantly correlated with other WM hubs as well as GM hubs signifying synchrony of BOLD signals between them. The strong correlations of the same WM hubs across slices suggests there are synchronous BOLD signal changes along WM tracts which possibly reflect motor or sensory information processing through the ascending and descending pathways. Strong correlations were also observed between neighboring WM hubs (within and between slices) which may indicate both tracts are involved in the same function viz. in our study dorsal column and spino-cerebellar ROIs were found to be strongly connected which corresponds to their neurobiological function as ascending pathways. However, these high correlations may also be affected by the venous drainage from GM hubs resulting into apparent functional connectivity. The strong correlation of WM hubs with neighboring GM hubs viz. dorsal column and spino-cerebellar tracts of WM with dorsal horns of GM suggests either a sharing of functional roles between them such as processing of sensory stimuli and transmitting them via ascending pathways, or potentially an effect of blood flowing from the GM through WM. It needs to be emphasized that BOLD signal is an indirect measure of neural activity based on haemodynamic changes and the potential for confounds from common or correlated vasculature could account for some of these effects. The precise biophysical basis of the BOLD changes seen in WM is still presently not understood. However, there is convincing evidence that drainage effects cannot explain the concomitant BOLD signals in WM in brain, and that WM BOLD signals may be induced by distant GM activity^16,17,20-22^. WM contains a large number of glial cells (mainly astrocytes and oligodendrocytes) that account for most of the tissue volume^46^. The glial cells are engaged in a number of functions including the regulation of blood flow and neurotransmitters, and presumably these functions affect the oxygenation and metabolic demands of WM^45,47^.

In order to evaluate the potential confounds from drainage effects we performed supplementary regression analyses. We found that regressing out signal from a WM hub can change the apparent connectivity between GM hubs or between GM and WM hubs, not only near it but also distant to it. These far away correlations are likely not driven by drainage from GM veins at such a distance. This shows that regressing out WM BOLD signal could lead to significantly erroneous calculations of correlation within and between WM and GM hubs. It therefore does not seem appropriate to use WM BOLD signals as nuisance regressors in fMRI studies of spinal cord.

From both intra and inter-slice analyses in our study, it was apparent that the variations of correlations between WM-WM, WM-GM and GM-GM are not random, but rather manifest a pattern suggesting that WM and GM hubs in the dorsal and dorso-lateral side exhibit greater temporal synchrony during a resting state than those in the ventral and ventro-lateral side. The dorsal horns of GM and the dorsal column and spino-cerebellar tracts of WM were also the regions to be most activated by tactile stimulation in our study. Thus, these results are consistent with the postulate that correlations during a resting state reflect networks that are jointly engaged during the response to a specific stimulus or task^14^.

There are limitations to this study. One drawback of ICA is that the criterion for selecting the number of components is not well defined and the optimal number of components is often application dependent. Here we came up with the desired number of components based on anatomical knowledge of WM tracts and empirical evidence of synchronized BOLD fluctuations in specific regions. Additionally, we found our main results were not affected by changes in the number of components used. Another limitation is the absence of an appropriate spinal cord atlas for squirrel monkeys, so we used hand drawn masks for WM tracts based on human and non-human spinal cord atlas^24,28-30^. Also, the animals of our study were anesthetized during image acquisition and it has been reported that anesthesia could influence the measurement of functional connectivity^48^. However, a high correspondence was observed in previous studies between connectivity measures of rsfMRI signals and local field potentials obtained under anesthesia conditions^6^ and thus it should not bias our results.

### Summary And Conclusion

Stimulus evoked BOLD signals were reliably detected in and around the dorsal column and spino-cerebellar tracts of WM of spinal cord using a GLM as well as model-free ICA method. The BOLD signal waveform was synchronous with the stimulus paradigm, and its spectrum showed peak power at the stimulus frequency and the amplitude of stimulus frequency was higher in the ascending tracts than the descending ones. ICA based decomposition of resting state BOLD signals showed coherent fluctuations from discrete WM regions which corresponded strongly with the anatomical location of white matter tracts of spinal cord. BOLD signals from these WM regions were strongly correlated with signals from other WM and GM hubs of the spinal cord during resting state in specific patterns that is consistent with the neurobiological function of WM tracts in spinal cord. Overall, these findings provide strong evidence of the presence of BOLD signal in the WM of spinal cord both in a resting state and under stimulus conditions that share many of the characteristics of signals used to assess functional connectivity in GM networks.

## Methods

### MRI data acquisition

A total of 22 adult male squirrel monkeys (Saimiri sciureus) were included in the study. During each MRI scan, the monkeys were anesthetized with isoflurane (0.8–1.2%) and mechanically ventilated, with head and body stabilized in a magnetic resonance (MR)-compatible frame. Vital signals (cardiac and respiratory cycles, core body temperature, end tidal CO2, peripheral capillary oxygen saturation SpO2 via pulse oximetry) were monitored and maintained within appropriate ranges throughout each imaging session. The respiration pattern during each fMRI scan was recorded and later used as a regressor in data processing. The procedures were performed under a protocol approved by the Institutional Animal Care and Use Committee at Vanderbilt University.

MRI scans were acquired on an Agilent 9.4T MRI spectrometer using a saddle-shaped transmit–receive surface coil (2.5 × 3 cm^2^ in size) positioned over each animal’s neck. High-resolution (0.25 × 0.25 mm^2^ in-plane resolution, 128 × 128 matrix) structural gradient echo images with magnetization transfer contrast (MTC) ([TR (repetition time)/TE (echo time): 220/3.24 ms] were acquired from the five contiguous slices covering C3-C7 segments following a gaussian radio frequency (RF) saturation pulse (flip angle, 820°; pulse width, 12 ms; RF offset, 5,000 Hz)].

FMRI data were also collected from the five contiguous slices covering the same geometry. Multiple runs (3 to 6) of stimulus evoked fMRI data (150 sequential volumes each run) were acquired using a block-design protocol (30 seconds ‘on’-30 seconds ‘off’ epochs). The stimulus comprised 8-Hz vibro-tactile stimulation applied to the distal finger pad of digit 2 of the dominant hand of each monkey delivered via a 2-mm-diameter round probe driven by an S88 Grass stimulator (Natus Neurology). Probes were in light contact with the skin during each baseline period. The rsfMRI data consisting of 300 sequential volumes were acquired using a fast gradient echo sequence (flip angle = ~ 18°, TR = 46.88ms, TE: 6.5ms, 3s per volume) with an in-plane resolution of 0.5 × 0.5 mm^2^ (64 × 64 matrix).

### FMRI data Pre-Processing and Analysis

All fMRI data underwent slice-by-slice, 2D motion correction implemented in MATLAB R2019a. Functional image volumes were first aligned using a 2D rigid body motion correction algorithm based on maximization of mutual information, by which three motion parameters were estimated (two translations and one rotation) slice by slice^6^. Motion parameters along with temporal signals extracted from muscle and CSF regions containing at least 70% of the cumulative variance (derived using principal components analysis) were considered nuisance parameters and regressed out using a GLM^2,4-6^. The rsfMRI signals were then corrected for physiological noise (respiratory signal) using RETROICOR^49^. The axial fMRI images were up sampled from 0.5 × 0.5 mm^2^ to 0.25×0.25mm to match the anatomic images. The rsfMRI signals were band-pass filtered (Chebyshev type2 MR filter, cut-off frequencies 0.01 and 0.1 Hz) prior to functional connectivity analyses.

Images of stimulus activation were obtained from 11 monkeys (61 runs). Analyses were performed on all voxels within the spinal cord of each subject using a GLM in SPM 12 software^50^. The stimulus pattern convolved with a standard HRF was used as a predictor to model the stimulus-driven signal changes and the GLM fit parameters (beta and contrast maps) were calculated. Group level analysis was performed using the individual monkey contrast maps to generate a map of significant signal changes (*p* < 0.05). GM and WM voxels were segmented using manually drawn gray and white matter masks. Maps of significant signal changes evoked by the stimuli were displayed on the GM and WM regions as statistical *t*-value maps (*t* threshold = 1) with a clustering criterion of a minimum of three continuous voxels. The time courses of significantly responsive voxels (thresholded at *t*> 1) from a selected ROI (for both GM and WM) were averaged across stimulus epochs (seven cycles in each run). We also calculated the percentage signal changes during stimulus ‘on’ periods with respect to the baseline signal which was a six-second window prior to the onset of each stimulus.

The fMRI data were registered to a customized spinal cord template using FSL to perform group analysis^51^. Spinal cord structural image template was generated based on MTC anatomic images from ten squirrel monkeys and used for registration. Initially, median fMRI images were coregistered to the structural MTC image in an individual subject space and the transformation was then applied to the fMRI time series data. In the second step each subject’s anatomic images were coregisterd to the spinal cord template and the transformation was applied to the fMRI data (both stimulus and resting runs) using FSL to perform the group analysis.

### Independent Component Analysis (ICA) of resting state fMRI data

Seed based correlation analyses have commonly been used to derive functional connectivity between two regions using BOLD signals^4,5^. However, seed-based analyses are limited by *a priori* hypotheses about which regions are engaged in a functional response and hence are not ideal for uncovering novel networks. Some previous studies have used ICA as a data driven technique to identify functional circuits in the GM of spinal cord^8,10,26,52^. Since we did not have prior knowledge of the location of functional circuits in WM of spinal cord, we chose ICA for unearthing them.

The coregistered 2D resting state functional data from the 22 monkeys (64 runs) were temporally concatenated, and group ICA was performed on the rsfMRI signals separately extracted from masked GM and WM voxels. Standard procedures in GIFT software^53^ were followed to obtain spatial ICA maps and their corresponding time series. Next, we used a dual regression technique to obtain subject-specific component maps, along with their associated time series^54^. In spatial ICA, each component denotes a region of coherent BOLD fluctuations spatially independent from other such regions. The GM and WM spatial component maps were thresholded (Z-score > 3 and Z-score > 4 respectively) and classified based on visual inspection of each component’s spatial profile. The thresholded WM spatial maps from ICA decompositions were compared with hand drawn WM tracts obtained from spinal cord atlas of human and non-human primates^24,28-30^.

For testing reliability and robustness of the WM ICs using GIFT software’s ICASSO method^32^, we ran a 45 component ICA 30 times using the FastICA algorithm. The algorithmic and statistical stabilities were investigated by running the algorithm many times with different initial values or/and with differently bootstrapped data sets.

### Functional Connectivity Measure

Functional connectivity between all the GM and WM ROIs obtained from ICA was computed at the individual subject as well as group level using the mean fMRI time series of each ROI. Connectivity was measured as the Pearson’s correlation coefficient (*r* value) between the time courses of pairs of ROIs and represented as a connectivity matrix.

### Regression Analysis on Functional connectivity between WM and GM hubs

The correlation values resulting from WM hubs can potentially be affected by drainage of veins from surrounding GM regions. Hence, we performed supplementary analysis to evaluate the effect of regressing out fMRI time course of individual WM and GM ROIs on the overall correlation between them. The analysis was done on the eroded masks of GM and WM ROIs of a representative slice (Slice 2) to account for the potential confounds in correlation values from vascular drainage effects and also partial volume effects.

### Network Analysis using Graph Theory

Network analysis was performed on the connectivity matrix using the Gephi software^35^ based on graph theory principles^36^. In graph theory, a network is defined by a collection of nodes (vertices), and links (edges) between pairs of nodes. Nodes in our study represent the WM and GM ROIs (from all the slices), while links represent correlation values between those ROIs. The graphical representation gives an idea of the strength of functional connectivity between the different ROIs with respect to their location in the spinal cord. We also computed the Node Strength, which represent the sum of the weight of the links connected to each node for both intraslice and interslice connections.
